# Examination of the Achillis-Jerk

**Published:** 1908-12-26

**Authors:** 


### Neurology.

### EXAMINATION OF THE ACHILLIS-JERK.

The Achillis-jerk is analogous to the knee-jerk, being obtained by percussing the tendo-Achillis in much the same way as the knee-jerk is obtained by percussing the ligamentum patellæ. Whereas, however, the knee-jerk is tested by every student when a nerve disease is in question, the Achillis-jerk is relatively seldom investigated. And yet there is increasing evidence to show that absence of Achillis-jerk means much the same as absence of knee-jerk, and that it may be detected the earlier of the two. It is surprising that absence of Achillis-jerk has hitherto attracted so little attention, seeing that its converse—namely, ankle clonus, is well enough known and understood. It is to Babinski that we owe the discovery that loss of Achillis-jerk may be as valuable a sign of locomotor ataxy as is loss of knee-jerk.

The best way of examining the Achillis-jerk is to have the patient kneeling up on a chair, facing the back of the chair to which he holds on with his hands whilst his legs from the knee downwards project straight behind him with the feet hanging loosely down beyond the chair-seat. The position is precisely that of a person kneeling upon a prie-dieu, except that the chair-seat is high enough to keep the feet quite free from the floor. It is important that the feet should not be held tense, but allowed to hang quite loose; to ensure this, the method of reinforcement may be required in exactly the same way as it sometimes is in testing the knee-jerks. The tendo-Achillis is then sharply struck with the percussion hammer; this hammer is almost essential in testing the Achillis-jerk, for without it one cannot be sure of the absence of this jerk, although when it is present it can often be obtained by percussing the tendo-Achillis with almost any fairly heavy hard thin object that may be handy, such as the ulnar edge of the open hand or the edge of a thin book or of a bed letter-case. If the Achillis-jerk is present a sharp jerk of the foot in the direction of plantar-flexion is observed.

If the patient is unable to kneel up on a chair in this way, there are two ways of examining the jerk with the patient lying down in bed. The better of the two is to have him prone upon his stomach, lying at full length, with the posterior aspects of the calves and feet towards one. Each foot is now taken up by the observer's left hand, the latter holding the foot gently across the instep and raising it until the leg is bent upon the thigh nearly to a right angle at the knee, whilst the foot is at right angles to the leg. The tendo-Achillis is now struck in the ordinary way with the percussion hammer held in the observer's right hand, and if the Achillis-jerk is present it can be felt by the observer's left hand even more readily than it can be seen.

Another method has to be employed when the patient cannot lie upon his abdomen. He is rolled on to his right side when the right Achillis-jerk is to be tested, and on to his left side for testing the left. In each case the leg that is not being tested is removed out of the way by being flexed up to a right angle at the hip. The other leg, which is undermost and in contact with the bed along its whole length, is kept in a straight line with the trunk at the thigh and at the same time flexed backwards to a right angle at the knee; the foot is at right angles to the leg, and kept flaccid, the toes and fore part of the foot being gently held in the observer's left hand whilst the tendo-Achillis is percussed in the usual way with the right.

All healthy persons have Achillis-jerks. People of nervous temperament have them exaggerated like the knee-jerks. In the diagnosis of locomotor ataxy the condition of the Achillis-jerk is almost if not quite as important as is that of the knee-jerk. Babinski holds the view that in tabes dorsalis the Achillis-jerk is usually lost before the knee-jerk. This is not an absolute rule. The Achillis-jerk is nearly always absent at the time the patient comes under observation, however, and sometimes it is absent when the knee-jerks are in the stage of being actually exaggerated. It is well known that although absence of knee-jerk and presence of Argyll-Robertson pupil together are pathognomonic of locomotor ataxia there are, on the one hand, a certain number of cases of Argyll-Robertson pupil that are not cases of tabes dorsalis, and, on the other hand, there are a certain number of cases of definite locomotor ataxy in which the knee-jerks are not lost. The diagnosis of locomotor ataxy, therefore, has sometimes to be made in the face of the presence of two knee-jerks. Dr. Byrom Bramwell's statistics put the proportion of such cases at over 10 per cent. of all cases of locomotor ataxy. The assistance that may be derived from examination of the Achillis-jerks in instances of this sort is well exemplified by one of Dr. Bramwell's cases in which the patient had suffered from typical lightning pains for eleven years, was markedly ataxic, and yet presented knee-jerks that were both present and very active; his Achillis-jerks were, however, both entirely absent even after reinforcement, and from this and from other points in the case there was no doubt as to the diagnosis.

